# Toward a “green” brain health agenda: establishing short- and long-term goals for the field of neurology in a changing climate

**DOI:** 10.3389/fneur.2025.1618365

**Published:** 2026-01-29

**Authors:** Esme D. Trahair, Trisha Dalapati, Danelle Levinson, Alexandra R. Linares, Aaron Reuben, Richard Bedlack

**Affiliations:** 1 Duke University School of Medicine, Durham, NC, United States; 2 Department of Medicine, Emory University, Atlanta, GA, United States; 3 Department of Psychology & Neuroscience, Duke University, Durham, NC, United States; 4 Department of Psychology, University of Virginia, Charlottesville, VA, United States; 5 Department of Neurology, Duke University, Durham, NC, United States

**Keywords:** brain health, climate change, environmental health, neuroepidemiology, neurology, population health, preventive medicine

## Abstract

The global burden of neurological disease is rising amid an aging population and accelerating climate change, yet environmental determinants of brain health remain underrecognized within neurology. Climate-related factors—including air pollution, extreme heat, environmental contaminants, and ecological disruptions—can contribute to neuroinflammation, cerebrovascular disease, neurodegenerative disorders, and mental health conditions. Development of climate-relevant short- and long-term goals within the field of neurology is in line with the discipline’s increasing interest in improving population brain health. This commentary categorizes “green” brain health priorities into five domains: (1) clinical practice, (2) public communication, (3) education and training, (4) research, and (5) policy. The recommendations put forth constitute an agenda that is relevant to many stakeholders, including professional societies, like the AAN. A dedicated commitment to environmental determinants of brain health is imperative to safeguarding global neurologic well-being in the face of an escalating climate crisis.

## 1 Introduction

As the planet warms and the global population ages (1, 2), the cumulative population health burden of neurological diseases is rising. Neurological disorders are now the *leading cause* of lost disability-adjusted life years globally, and they are second only to cardiovascular diseases as a cause of premature mortality (3–5). To address this, the American Academy of Neurology (AAN) (6), World Health Organization (WHO) (4), US Centers for Disease Control and Prevention (CDC) (7), and other influential health organizations have proposed initiatives to foster population-level brain health across the globe. However, the roles of climate change and environmental health are not prioritized in these agendas. Given the extraordinary risks posed by climate change, it is imperative that our field: (1) formulates a shared position on what is known about the potential interplay of climate change with brain health, and (2) establishes short- and long-term climate-relevant goals for protecting brain health.

This commentary, inspired by an interdisciplinary health professions pilot course at Duke University School of Medicine, asserts that neurology trainees, practitioners, and professional organizations can *and must* steer our field to improve global population neurologic health amid a changing climate crisis. After defining the central concepts – *brain health* and *climate change* – and examining their intersection, we outline a proposed agenda of “green” brain health priorities, categorized into five key domains: (1) clinical practice, (2) public communication, (3) education and training, (4) research, and (5) policy. We propose that these recommendations are imperative to executing the existing commitments to brain health voiced by several stakeholder organizations given the current global burden of neurologic diseases and advancement of climate change.

## 2 Defining brain health

The term *brain health* first appeared in academic literature in 1989. However, it was not until 2006 that the first comprehensive, evidence-based definition was published by the National Institutes of Health (NIH) Cognitive and Emotional Health Project (4, 8, 9). The NIH proposed:

The development and preservation of the multidimensional cognitive structure that allows the older adult to maintain social connectedness, an ongoing sense of purpose, and the abilities to function independently, to permit functional recovery from illness or injury, and to cope with functional deficits (9).

Since then, interest in “brain health” has expanded significantly, from fewer than five citations in the academic literature in 2006 to more than 1,800 by 2021 (4, 8). The most recent attempt to create a unified definition of brain health comes from the AAN’s 2024–2028 Strategic Plan:

Brain health is a continuous state of attaining and maintaining the optimal neurologic function that best supports one’s physical, mental, and social well-being through every stage of life (6).

This definition and further explication from the Academy about its formulation reveal several fundamental components of brain health.

1. *Disease state:* Brain health does not imply the absence or presence of neurological disease; everyone has brain health (4, 6, 10–12).
2. *Life stage:* Brain health is not limited to a specific stage of life; it refers to brain function across the lifespan (4, 6–8, 11, 12).
3. *Optimization:* Optimization is a relative, personalized concept; optimal brain health for one individual may look very different from that of someone else (4, 6, 8, 12).
4. *Context:* Brain health is experienced in the holistic context of one’s life; it is a function of biomedical, psychological, sociocultural, economic, and environmental factors (4, 8–10, 12, 13).
5. *Prevention:* On a population level, advances in the field of brain health necessitate increased prevention efforts and attention to upstream drivers of brain health (6, 8, 10, 12–14).

## 3 The interplay of climate change with brain health

Definitions for “climate change” vary based on audience (e.g., academic versus public) and context (e.g., political versus ecological) (15). This commentary defines “climate change” as long-term anthropogenic shifts in global temperatures and weather patterns, resulting in impacts both direct (e.g., rising ocean levels, extreme weather events) and indirect (air and water pollution, environmental degradation) (16–18). In the context of brain health, climate change can be contextualized within a broader *neural exposome* – which encompasses an integrated array of exogenous and endogenous non-genetic factors that impact neurologic health and illness (19–21). Along with other components of the neural exposome, climate change contributors, including environmental contaminants, noise and light pollution, and weather patterns, exert influence on an individual’s health starting before conception, and through the life course they impact neuroplastic mechanisms that underpin major neurologic illness (22–24). A robust body of literature has demonstrated that climate change and its drivers have a wide range of negative health consequences, including but not limited to worsening of heat-related illnesses, malnutrition, vector-borne infections, and psychiatric illness, via direct health effects as well as indirect and downstream disruptions to systems of care and healthcare access (16–18).

### 3.1 Neurologic effects of climate change and its contributors

Some of the most well-defined climate risks that may plausibly harm brain health include air pollution, ecological changes (e.g., rising sea levels, altered animal migration patterns, destruction of green space), rising temperatures, and extreme weather events. While by no means a comprehensive list, these climate change contributors are well-established as contributors to poor neurologic outcomes via mechanisms affecting neurogenesis and neuroplasticity (20, 22, 24). Table 1 demonstrates the proposed mechanisms of neurologic harm and the resultant neurologic disorders that have been linked to selected aspects of climate change. Furthermore, the neurologic burden of climate change is not experienced equally across the human population. Social and environmental drivers of health, inequitable resource allocation ranging from local to international scales, and variable healthcare access perpetuate structures and ecosystems through which historically marginalized and disenfranchised populations disproportionately experience (1) direct neurotoxic environmental exposures and (2) downstream effects of climate change with brain health consequences (17, 18, 24, 25).

**Table 1:** Selected examples of neurologic effects of climate change and its contributors.

Example	Phenomena	Mechanisms of harm	Brain diseases
Environmental contaminants – heavy metals, pesticides, air pollutants	Increased environmental deposition due to human production and consumption (16) Altered distribution due to changes in weather patterns (16)	Abnormal neuronal development during gestation due to placental transmission (22)	ASD, childhood behavioral & cognitive disorders (22, 51)
		Developmental toxicity affecting molecular and functional neuroplasticity (24)	PD, AD, ALS (24, 51)
Natural disasters – droughts, floods, and other extreme weather events	Increased frequency due to ecologic shifts like elevated sea levels and rising temperatures (16) Lack of infrastructure in areas that are newly vulnerable to natural disasters (17)	Stress secondary to downstream effects of weather events (displacement, poverty, loss of human life) causing inhibited neurogenesis (23)	Depression, anxiety, PTSD (16)
		Secondary malnutrition during early development inhibiting several formative neuroplastic processes (23)	Neuropsychiatric illnesses, ADHD (16)
Rising average and extreme temperatures – “global warming”	Increased human consumption of fossil fuels and release of greenhouse gases (50) Deforestation, agriculture practices, and industrialization (50)	Altered brain metabolism, reduction in cerebral blood flow, damaged blood–brain and blood-CSF barriers, changes in gene expression (16, 23)	Migraines, seizure, epilepsy (5, 16, 23)
		Altered animal migration patterns causing increases vector-borne, zoonotic, and water-borne diseases (16)	Infectious neurotropic diseases – Zika, Ebola, Naegleria (16)

ASD, autism spectrum disorder; PD, Parkinson’s disease; AD, Alzheimer’s disease; ALS, amyotrophic lateral sclerosis; PTSD, post-traumatic stress disorder; ADHD, attention deficit hyperactivity disorder.

### 3.2 Brain health and climate change: a reciprocal relationship

Apart from what is already known about the negative effects of climate change on population brain health, there is an additional consideration specific to *neurological* illnesses. Brain function is a crucial component of an individual’s and population’s experience of disease and ability to respond to environmental challenges in the short- and long-term. Therefore, a reciprocal relationship exists between compromised brain function and worsening climate change (3, 4, 11). This interdependence is the basis for the theory of “green brain capital,” which emphasizes that healthy brains are needed to maintain a healthy environment *and* vice versa (11, 26). Therefore, by improving population brain health, we may also maximize our collective cognitive resources to fight climate change.

## 4 A call to action

Organizations with existing commitments to brain health, including the AAN and WHO, have considerable influence over a wide audience of interdisciplinary population health stakeholders, including clinical practitioners, public health professionals, policymakers, and the public. Their current positions establish strong foundations for incorporating a comprehensive set of goals for optimizing brain health in our rapidly changing climate, as is evidenced by the two following position statements—official, evidence-based documents developed by a coalition of expert professionals to inform policy and best practices on an advocacy issue.

### 4.1 The AAN 2021 telehealth position statement

The AAN regularly publishes position statements to influence and inform neurologic clinical practice, public policy, and ethics (27). The AAN position statement on telemedicine, last updated in 2021, advocates for expanded access to care, insurance coverage, provider reimbursement, and research on the optimization/limitations of virtual care (28). These efforts have measurably influenced public policy – in 2023, the AAN’s advocacy efforts contributed to extended Medicare coverage of telehealth services through the end of 2024 (29). The tangible impacts of the AAN’s advocacy for investment in and expansion of neurology telehealth services make clear that comparable efforts by the AAN and its peer organizations on the subject of green brain health would drive meaningful change in clinical practice and policy.

### 4.2 The American Lung Association 2022 healthy air position

The American Lung Association (ALA) has a longstanding record of advocating for environmental health initiatives and lobbying for policies that reduce pollution and mitigate climate change (30). These efforts have helped fortify the Clean Air Act and effectuate changes in the Environmental Protectional Agency’s policies to regulate emissions from motor vehicles, power plants, and gas and oil operations (31). In addition, the ALA has partnered with the CVS Health Foundation on an intervention in Phoenix, Arizona, which offers tools to healthcare providers and patients to reduce health risks on days of poor air quality and promotes local policies to improve lung health (32). The ALA’s model of research, advocacy, public communication, education, and clinical practice provides a meaningful example for how neurology professional societies could influence environmental health.

## 5 Discussion

**Figure 1:**
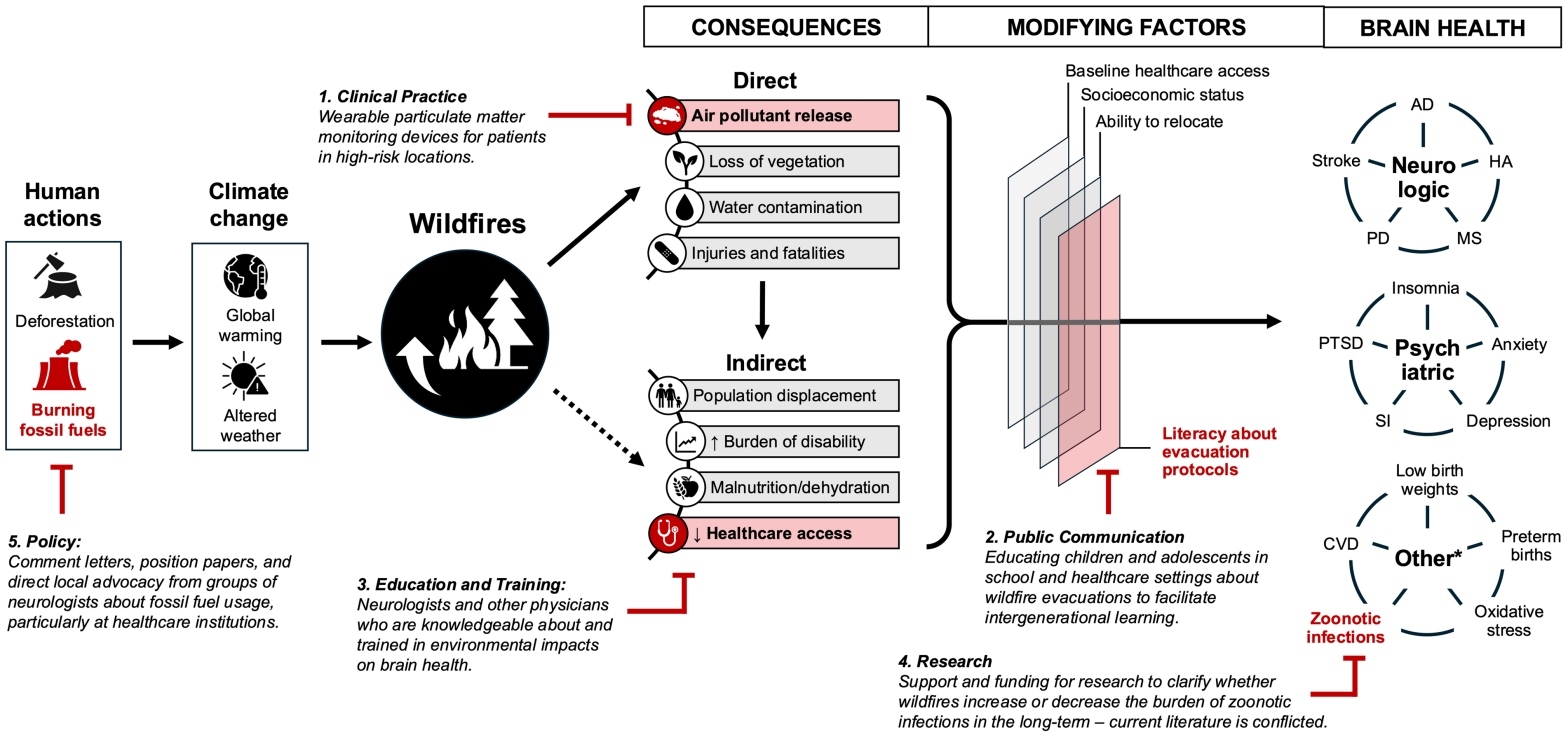
Examples of green brain health interventions on wildfire neurotoxicity. A schematic that illustrates a quasi-linear framework of the progression from long-term anthropogenic changes to downstream neurological health effects. Five potential intervention points that correspond with the priority domains established in Table 2 are illustrated (16, 17, 52). *Other illnesses with neurologic outcomes. AD, Alzheimer’s disease; HA, headache; MS, multiple sclerosis; PD, Parkinson’s disease; PTSD, post-traumatic stress disorder; SI, suicidal ideation; CVD, cardiovascular disease.

The neurologic burden of climate change is significant and growing (2, 3, 25). Exposures to the direct impacts (rising sea levels, natural disasters, elevated temperatures) and indirect impacts (environmental pollution and ecologic changes) of climate change, particularly in the prenatal period and first 2 years of life, lead to brain disease through a variety of neurotoxic mechanisms (16, 20, 22, 23). Considering the major morbidity and mortality associated with these adverse neurologic consequences, we propose an initial green brain health agenda that would optimize long-term population brain health that spans five interrelated domains: clinical practice, communication, education and training, research, and public policy (Table 2). This agenda is a starting point that can be developed further through stakeholder engagement and discussion in the field; case studies are illustrated in Figure 1.

**Table 2:** Proposed priorities for a green brain health agenda.

Domain	Priorities
**Clinical practice**	Develop protocols to assess and screen neurotoxic climate exposures in patients. Empower patients with accessible resources and information to influence their own green brain health.
**Public communication**	Relay information about green brain health best practices to the public with public awareness campaigns. Develop green brain health content for maternal-fetal medicine and pediatric neurology settings to facilitate intergenerational learning.
**Education & Training**	Incorporate information about neurological impacts of climate change in didactic medical school pathophysiology curricula. Establish accredited preventive neurology fellowship programs which emphasize green brain health.
**Research**	Identify and prioritize research on detrimental climate exposures with the highest neurologic mortality and morbidity impacts. Review and reallocate funding to preventive brain health research. Monitor and investigate climate mitigation and adaptation responses for adverse neurological effects.
**Policy**	Publish academic society and medical institutional statements detailing green health positions, priorities, and commitments. Advocate for green brain health priorities in neurology professional societies.

### 5.1 Clinical practice: screenings and patient resources

In line with the preventive nature of brain health, neurology clinical practice should include evidence-based routine protocols to assess and screen individual patient-level climate exposures that may pose neurological risk (33, 34). These tools can be incorporated into routine office visits along with traditional sociodemographic screenings, and they should have clear follow-up steps in the event of positive screens, focused on diminishing harmful exposures that have been identified. For example, if a patient screens positive for elevated household air pollution based on screening questions, they should receive education and recommendations about minimizing exposures through improved household ventilation and ways to improve air filtration (35). Providers should also be knowledgeable about patient-centered resources to improve green brain health. Existing tools like the AirNow Air Quality Index calculator and forecast (36), or Beat the Heat in Houston interactive map (37), are good models for tools that can help patients improve and maintain their brain health.

### 5.2 Public communication: awareness campaigns and pediatric education

Brain health, and specifically *green* brain health, is an appropriate and promising topic for public awareness campaigns. A similar model to effective stroke awareness campaigns (e.g., Stroke Heroes Act FAST) can be used to disseminate important information about green brain health to a large, diverse audience (38, 39). In addition, green brain health education should be prioritized in reproductive health, maternal-fetal medicine, and pediatric settings. The pre-conception period (including pregnancy planning), gestation, and the first 2 years of life comprise of several critically sensitive periods of neuroplasticity in which one’s neural exposome exposures are most deleterious, with potentially long-lasting consequences on brain health (12, 22, 40). Moreover, pediatric education facilitates intergeneration learning, where caregivers will indirectly receive information about green brain health (41, 42).

### 5.3 Education & Training: undergraduate medical curricula and fellowship training

More and more medical schools are incorporating information about planetary health and climate change into their curricula, both woven into existing material and as a dedicated topic (43, 44). It is critical that students learn about green brain health and the implications of climate change for neurological wellness and illness. Existing proposed curricula are available to be replicated, and they demonstrate the feasibility of either (1) a dedicated course to climate change and medicine during undergraduate medical education or (2) incorporating climate-relevant information into the existing curricula (e.g., asking educators to include at least one slide on the environmental implications of the medical content of their lectures) (45–47). At the graduate level, an accredited preventive neurology fellowship program should be established to train subspecialists in green brain health, among other preventive neurology topics (10, 14).

### 5.4 Research: literature review, funding, and implementation evaluation

Literature detailing the adverse effects of climate change on neurological wellbeing needs to be published, consolidated, and reviewed to identify top priority pathologies, brain functions, and populations. Institutions and grant-making organizations should favor funding preventive brain health research as a long-term investment in population brain health. Multidisciplinary, interprofessional groups are best equipped to conduct high impact research that appropriately integrates exogenous, endogenous, and behavioral factors of the neural exposome. Finally, emerging interventions and new techniques designed to improve green brain health need to be monitored not only for their impact but also for unintended negative consequences (16).

### 5.5 Policy: advocacy and positions

Professional societies are an effective, realistic channel for practitioner and researcher advocacy (48). On an institutional basis, neurological academic societies and medical institutions should routinely publish statements emphasizing the importance of and their commitment to green brain health to establish collective expert opinion about policy priorities (48, 49). Furthermore, it is imperative policy recommendations at all levels (local, national, global) recognize that brain and mental health disorders are an ongoing global public health crisis that affects nearly half of the world’s population and will realistically continue to do so in the decades to come, even with appropriate interventions (25). These statements would also serve to standardize and publicize evidence-based findings and standards of practice for practitioners to be aware of.

## 6 Conclusion

Climate change has serious implications for all areas of human health, and its effects on the brain and nervous system warrant especially urgent consideration given its unique, reciprocal bearings on society’s ability to control and adapt to a changing climate. Stakeholders should capitalize on escalating academic and professional interest in brain health to establish clear, unified goals for the field. While the recommendations in this commentary span five different domains, they constitute an agenda that largely focuses on preventive brain health, views neurologic health as a lifelong pursuit (with critical developmental windows in the prenatal period and early infancy), and emphasizes interprofessional collaboration in addressing this inherently multidisciplinary challenge. The priorities presented for neurological clinical practice, public communication, education and training, research, and policy are starting points for future efforts and interventions to preserve and protect green brain health for all.

## Data Availability

The original contributions presented in the study are included in the article/supplementary material, further inquiries can be directed to the corresponding author.

## References

[1] Martin JB. The integration of neurology, psychiatry, and neuroscience in the 21st century. Am J Psychiatry. (2002) 159:695–704. doi: 10.1176/appi.ajp.159.5.695, 11986119

[2] Béjot Y Yaffe K. Ageing population: a neurological challenge. Neuroepidemiology. (2019) 52:76–7. doi: 10.1159/000495813, 30602150

[3] Sisodiya SM. Climate change and the brain. Brain. (2023) 146:1731–3. doi: 10.1093/brain/awad076, 36941776 PMC10151177

[4] WHO Mental Health and Substance Use Team. Optimizing brain health across the life course: WHO position paper. Geneva: World Health Organization (2022).

[5] WHO Mental Health and Substance Use Team. Intersectoral global action plan on epilepsy and other neurological disorders: 2022–2031. Geneva: World Health Organization (2023).

[6] Rost NS Salinas J Jordan JT Banwell B Correa DJ Said RR . The brain health imperative in the 21st century—a call to action: the AAN brain health platform and position statement for the American Academy of Neurology’s committee on public engagement. Neurology. (2023) 101:570–9. doi: 10.1212/WNL.0000000000207739 37730439 PMC10558159

[7] CDC Healthy Brain Initiative. Promoting brain health. Atlanta, GA: Centers for Disease Control and Prevention (2011).

[8] Chen Y Demnitz N Yamamoto S Yaffe K Lawlor B Leroi I. Defining brain health: a concept analysis. Int J Geriatr Psychiatry. (2022) 37. doi: 10.1002/gps.5564 34131954

[9] Hendrie HC Albert MS Butters MA Gao S Knopman DS Launer LJ . The NIH cognitive and emotional health project: report of the critical evaluation study committee. Alzheimers Dement. (2006) 2:12–32. doi: 10.1016/j.jalz.2005.11.004, 19595852

[10] Sabayan B Doyle S Rost NS Sorond FA Lakshminarayan K Launer LJ. The role of population-level preventive care for brain health in ageing. Lancet Healthy Longev. (2023) 4:e274–83. doi: 10.1016/S2666-7568(23)00051-X, 37201543 PMC10339354

[11] Eyre HA Meidl RA. Brain capital is key to a sustainable future. Houston, TX: Houst Rice Univ Bak Inst Public Policy (2023).

[12] Owolabi MO Leonardi M Bassetti C Jaarsma J Hawrot T Makanjuola AI . Global synergistic actions to improve brain health for human development. Nat Rev Neurol. (2023) 19:371–83. doi: 10.1038/s41582-023-00808-z, 37208496 PMC10197060

[13] Avan A Hachinski V Brain Health Learn and Act Group. Brain health: key to health, productivity, and well-being. Alzheimers Dement. (2022) 18:1396–407. doi: 10.1002/alz.12478 34569702

[14] Sabayan B Isaacson R Rost N. Opinion & special article: preventive neurology: an emerging field toward brain health. Neurology. (2021) 97:916–9. doi: 10.1212/WNL.0000000000012554 34315783 PMC8601209

[15] Lineman M Do Y Kim JY Joo GJ. Talking about climate change and global warming. PLoS One. (2015) 10:e0138996. doi: 10.1371/journal.pone.0138996, 26418127 PMC4587979

[16] Ruszkiewicz JA Tinkov AA Skalny AV Siokas V Dardiotis E Tsatsakis A . Brain diseases in changing climate. Environ Res. (2019) 177:108637. doi: 10.1016/j.envres.2019.108637, 31416010 PMC6717544

[17] Haines A Ebi K. The imperative for climate action to protect health. N Engl J Med. (2019) 380:263–73. doi: 10.1056/NEJMra1807873 30650330

[18] Patz JA Frumkin H Holloway T Vimont DJ Haines A. Climate change: challenges and opportunities for Global Health. JAMA. (2014) 312:1565–80. doi: 10.1001/jama.2014.13186, 25244362 PMC6108836

[19] NIH National Institute of Neurological Disorders and Stroke. The neural exposome top priorities working group: report to the national advisory neurological disorders and stroke council. Bethesda, MD: National Institutes of Health (2024).

[20] Tamiz AP Koroshetz WJ Dhruv NT Jett DA. A focus on the neural exposome. Neuron. (2022) 110:1286–9. doi: 10.1016/j.neuron.2022.03.019, 35349785

[21] Zhang P Carlsten C Chaleckis R Hanhineva K Huang M Isobe T . Defining the scope of Exposome studies and research needs from a multidisciplinary perspective. Environ Sci Technol Lett. (2021) 8:839–52. doi: 10.1021/acs.estlett.1c00648, 34660833 PMC8515788

[22] Scher MS. Interdisciplinary fetal-neonatal neurology training applies neural exposome perspectives to neurology principles and practice. Front Neurol. (2024) 14:1321674. doi: 10.3389/fneur.2023.1321674 38288328 PMC10824035

[23] Kunikullaya UK. An integrated approach to understanding the effects of exposome on neuroplasticity. Behav Brain Res. (2025) 485:115516. doi: 10.1016/j.bbr.2025.115516 40024484

[24] Bain L Norris SP Stroud C, editors. Environmental neuroscience: advancing the understanding of how chemical exposures impact brain health and disease: proceedings of a workshop [Internet]. Washington, DC: National Academies Press; 2020 33180402

[25] Winkler AS Gupta S Patel V Bhebhe A Fleury A Aukrust CG . Global brain health—the time to act is now. Lancet Glob Health. (2024) 12:e735–6. doi: 10.1016/S2214-109X(23)00602-2, 38493787

[26] Ibanez A Eyre H. Brain capital, ecological development and sustainable environments. BMJ Ment Health. (2023) 26:e300803. doi: 10.1136/bmjment-2023-300803, 37832976 PMC10603528

[27] American Academy of Neurology. AAN policy on position statement topics. Washington, DC: American Academy of Neurology (2024).

[28] Hatcher-Martin JM Busis NA Cohen BH Wolf RA Jones EC Anderson ER . American Academy of Neurology telehealth position statement. Neurology. (2021) 97:334–9. doi: 10.1212/WNL.0000000000012185, 33986141 PMC8377877

[29] American Academy of Neurology. 2023 advocacy year in review. Minneapolis, MN: The American Academy of Neurology (2024).

[30] American Lung Association. Healthy air. Chicago, IL: American Lung Association (2022).

[31] American Lung Association. 2024 Help is on the way: clean air protections across the finish line. Available online at: https://www.lung.org/policy-advocacy/healthy-air-campaign/dont-delay-clean-air-today (Accessed Nov 26, 2024)

[32] American Lung Association. American Lung Association and CVS Health Foundation collaborate to address and educate communities on health impacts of poor air quality. New York, NY: PR Newswire (2024).

[33] Castellani B Bartington S Wistow J Heckels N Ellison A Van Tongeren M . Mitigating the impact of air pollution on dementia and brain health: setting the policy agenda. Environ Res. (2022) 215:114362. doi: 10.1016/j.envres.2022.114362, 36130664

[34] Schumann G Barciela R Benegal V Bernard A Desrivieres S Feng J . The earth, brain, health commission: how to preserve mental health in a changing environment. Nature Mental Health. (2024) 2:1121–3. doi: 10.1038/s44220-024-00314-1

[35] Hadley MB Baumgartner J Vedanthan R. Developing a clinical approach to air pollution and cardiovascular health. Circulation. (2018) 137:725–42. doi: 10.1161/CIRCULATIONAHA.117.030377, 29440198 PMC5950725

[36] EPA and Partners. AirNow.gov—Home of the U.S. Air Quality Index. London: AirNow (2024).

[37] ArcGIS, National Center for Atmospheric Research. 2024. Beat the heat in Houston. Available online at: https://ncar.maps.arcgis.com/apps/PublicInformation/index.html?appid=9d69e48135554580a25fed921c1a4d6b. (Accessed May 10, 2024)

[38] Rasura M Baldereschi M Di Carlo A Di Lisi F Patella R Piccardi B . Effectiveness of public stroke educational interventions: a review. Eur J Neurol. (2014) 21:24102755:11–20. doi: 10.1111/ene.12266 24102755

[39] Wall HK Beagan BM O’Neill HJ Foell KM Boddie-Willis CL. Addressing stroke signs and symptoms through public education: the stroke heroes act FAST campaign. Prev Chronic Dis. (2008) 5:A49. 18341784 PMC2396980

[40] Farina FR Bridgeman K Gregory S Crivelli L Foote IF Jutila OEI . Next generation brain health: transforming global research and public health to promote prevention of dementia and reduce its risk in young adult populations. Lancet Healthy Longev. (2024) 5:100665. doi: 10.1016/j.lanhl.2024.100665 39718180 PMC11972554

[41] Lawson DF Stevenson KT Peterson MN Carrier SJ Strnad R Seekamp E. Children can foster climate change concern among their parents. Nat Clim Chang. (2019) 9:458–62. doi: 10.1038/s41558-019-0463-3

[42] Boudet H Ardoin NM Flora J Armel KC Desai M Robinson TN. Effects of a behaviour change intervention for girl scouts on child and parent energy-saving behaviours. Nat Energy. (2016) 1:16091. doi: 10.1038/nenergy.2016.91

[43] Howard B. Climate change in the curriculum. Washington, DC: AAMC news (2019).

[44] Sullivan JK Lowe KE Gordon IO Colbert CY Salas RN Bernstein A . Climate change and medical education: an integrative model. Acad Med. (2022) 97:188–92. doi: 10.1097/ACM.0000000000004376, 34432714

[45] Philipsborn RP Sheffield P White A Osta A Anderson MS Bernstein A. Climate change and the practice of medicine: essentials for resident education. Acad Med. (2021) 96:355–67. doi: 10.1097/ACM.0000000000003719, 32910006

[46] Dalapati T Nick SE Chari TA George IA Hunter Aitchison A MacEachern MP . Interprofessional climate change curriculum in health professional programs: a scoping review. Educ Sci. (2023) 13:945. doi: 10.3390/educsci13090945

[47] Rabin BM Laney EB Philipsborn RP. The unique role of medical students in catalyzing climate change education. J Med Educat Curri Develop. (2020) 7:2382120520957653. doi: 10.1177/2382120520957653, 33134547 PMC7576899

[48] Marty-Chastan C Sherman J. Medical professional societies are well positioned to work toward climate change reform in practice and policy. New York, NY: The Commonwealth Fund (2024).

[49] The Medical Society Consortium on Climate & Health. Annual report 2023. Fairfax, Virginia: George Mason University Center for Climate Change Communication (2023).

[50] Rahimpour M Farsi M Makarem M. CO_2_ emission sources, greenhouse gases, and the global warming effect In: Advances in carbon capture. Shiraz, Iran: Woodhead Publishing (2020). 3–28.

[51] Yu G Wu L Su Q Ji X Zhou J Wu S . Neurotoxic effects of heavy metal pollutants in the environment: focusing on epigenetic mechanisms. Environ Pollut. (2024) 345:123563. doi: 10.1016/j.envpol.2024.123563, 38355086

[52] Myers SS. Planetary health: protecting human health on a rapidly changing planet. Lancet. (2017) 390:2860–8. doi: 10.1016/S0140-6736(17)32846-5 29146123

